# Photoreceptor-like cells from reprogramming cultured mammalian RPE cells

**Published:** 2013-05-30

**Authors:** Run-Tao Yan, Xiumei Li, Jian Huang, Clyde Guidry, Shu-Zhen Wang

**Affiliations:** 1 Department of Ophthalmology; University of Alabama at Birmingham School of medicine, Alabama; 2 Department of Medicine; University of Alabama at Birmingham School of medicine, Alabama

## Abstract

**Purpose:**

Previous studies showed that chick retinal pigment epithelium (RPE) cells can be reprogrammed by a specific gene to take on the path of photoreceptor differentiation. In this study, we tested whether this reprogramming scheme could be applied to mammalian RPE cells.

**Methods:**

Human RPE cell lines ARPE-19, a spontaneously transformed line of RPE cells derived from a 19-year-old person, and hTERT-RPE1, a telomerase-immortalized RPE cell line derived from a 1-year-old person, were commercially obtained and cultured as recommended. Primary RPE cell cultures were established using RPE isolated from 3- to 6-month-old pig and postnatal day 5 mouse. Cultured cells were transduced with a virus expressing *neuroD*, *neurogenin1* (*ngn1*), or *ngn3*, *basic helix-loop-helix* (bHLH) genes previously identified as capable of inducing RPE-to-photoreceptor reprogramming in the chick system. Alternatively, cells in the culture were transfected chemically or physically through electroporation with vector DNA expressing one of the three genes. The cultures were then analyzed for RPE-to-photoreceptor reprogramming with in situ hybridization and/or immunostaining for photoreceptor gene expression.

**Results:**

Both hTERT-RPE1 and ARPE-19 cultures gave rise to cells bearing markers of photoreceptors after transduction or transfection with vehicles expressing *neuroD* or *ngn1*. The new cells expressed genes encoding photoreceptor proteins, including interphotoreceptor retinoid-binding protein IRBP), recoverin, retinal cone arrestin 3, transducin α-subunit, Cone-rod homeobox protein (Crx), and red opsin. They displayed morphologies resembling differentiating photoreceptor cells. In primary porcine and mouse RPE cell cultures, transduction with lenti virus (Lvx-IRES-ZsGreen1) expressing *ngn1* or *ngn3* resulted in the emergence of ZsGreen1^+^ cells that exhibited morphologies reminiscent of differentiating photoreceptor cells. Immunochemistry showed that some ZsGreen1^+^ cells were positive for neural marker microtubule-associated protein 2 (Map2) and photoreceptor hallmark proteins red opsin and rhodopsin.

**Conclusions:**

The results suggest that cells in human RPE cell lines and in primary cultures of porcine and mouse RPE respond to gene-induced reprogramming by giving rise to photoreceptor-like cells. The responsiveness of primary RPE cells, especially those from porcine cells, enhances the biologic feasibility of exploring RPE-to-photoreceptor reprogramming for in situ mammalian photoreceptor replacement without cell transplantation.

## Introduction

Photoreceptor degeneration leads to blindness because no effective interventions are available. One of the promising therapies on the scientific horizon is photoreceptor replacement. Studies in mice have demonstrated successful photoreceptor replacement [1,2], but applying this technology to humans is a challenge due to a lack of a viable source of transplantable differentiating photoreceptors or their precursors [3,4].

Investigative approaches to producing differentiating photoreceptor cells currently highlight the use of embryonic stem cells and induced pluripotent stem cells. Significant advancement and exciting outcomes have been obtained. Nonetheless, naturally occurring regeneration, such as wound healing, involves awakening cells at or near a wound site to produce, in vivo and in situ, new cells needed to heal the wound. This in vivo cell regeneration offers advantage for cell replacement therapies as it avoids cell transplantation and associated risk and complications. Unfortunately, in vivo cell regeneration remains unattainable for various degenerative diseases, including photoreceptor degeneration, due to a lack of an explicit regeneration mechanism in mammals.

One way to circumvent this barrier is to tweak a nearby tissue capable of wound healing in such a manner that the tissue functions as a source of the desirable cell. For in situ photoreceptor regeneration, the RPE may offer potential. Besides its convenient location, RPE possesses two known properties: proliferation and plasticity. Under normal conditions, a small population of cells in the periphery proliferates while most RPE cells remain quiescent [5]. However, RPE cells proliferate substantially under disease conditions [6-8], after retinal detachment [9-11], or when stimulated physically [12]. A recent study showed that ~10% of RPE cells isolated from adult human exhibit “stem cell-like” properties and can re-enter the cell cycle once in culture [13]. RPE proliferation may result in RPE regeneration/wound healing [14-18] and/or retinal detachment when progeny cells differentiate into cells with tractional force [19], leading to vision impairment.

With mounting knowledge on the regulatory guidance of photoreceptor genesis during retinal development, an alternative approach to produce new photoreceptor cells has emerged —reprogramming the RPE by genes with pro-photoreceptor activities, thereby channeling RPE proliferation and plasticity toward photoreceptor production. Previous studies using the chick system tested a number of genes hypothesized or implicated in the regulatory hierarchies of retinogenesis or photoreceptor genesis and identified several *basic helix-loop-helix (*bHLH) genes, including *neuroD*, *neurogenin1* (*ngn1*), and *ngn3*, as effective inducers of RPE-to-photoreceptor reprogramming [20-22]. For instance, as many as 80% of the cells in a primary RPE cell culture derived from day 6 chick embryos show detectable photoreceptor traits with reprogramming by *ngn1* or *ngn3* [22]. Reprogrammed cells express an array of photoreceptor genes and exhibit photoreceptor morphologies. Perhaps more importantly, reprogrammed cells show physiologic properties that are hallmarks of photoreceptor cells: response to light and to 9-cis-retinal [22,23]. RPE-to-photoreceptor reprogramming also commences in vivo in the embryonic chick eye when reprogrammed by *ngn3* [24].

As a step in studying whether this RPE-to-photoreceptor reprogramming might bear clinical implication, we tested it with human RPE cell lines and primary RPE cell cultures derived from postnatal mouse and 3–6-month-old pig. Here we report the production in these mammalian RPE cell cultures of cells bearing similarities to young photoreceptor cells.

## Methods

### Generating gene expression cassettes

Human *neuroD* coding sequence was reverse transcriptase (RT)-PCR amplified, cloned into pGEM-T (Promega, Madison, WI), and its sequence verified. The *neuroD* sequence was then inserted into a replication-deficient retroviral vector, pMSCV (Clontech, Mountain View, CA), and the recombinant DNA (MSCV-neuroD) was transfected into packaging cells to produce retrovirus. Retrovirus particles were harvested from the cell culture medium by two rounds of centrifugation, with the first to remove cells and cell debris (high speed centrifuge) and the second to pellet the viral particles (ultra centrifuge) for concentration, following the protocol provided by the manufacturer. MSCV-expressing GFP (MSCV-GFP) was generated and used as a control.

To construct *ngn1* expression cassettes, the coding region of human *ngn1* was PCR amplified and cloned into pGEM-T. After sequence verification, the DNA was subcloned into retroviral vector pMSCV and adeno-associated viral (AAV) vector pAAV (Stratagene) modified to include the CAG sequence for high level of gene expression [25]. Human *ngn1* was also subcloned into the lenti-viral vector pLvx-IRES95 ZsGreen1 (Clontech), which allows the simultaneous expression of Ngn1protein and a green fluorescent protein (Zsgreen1). The recombinant DNA was cotransfected with a mixture of plasmids that respectively express viral proteins needed for producing viral particles in packing cells (Lenti-X 293T), following manufacturer’s procedure. Culture medium contained viral particles released from the packing cells were harvested and store at -80 °C until use. Lenti-virus expressing human *ngn3* (Lvx-ngn3-IRES-ZsGreen1) was similarly produced.

### Human RPE cell line culture

Human RPE cell line ARPE-19 was obtained from the American Type Culture Collection. ARPE-19 cells were transfected with AAV-ngn1 DNA using Fugene 6 (Roche, Penzberg, Germany), following the steps recommended by the manufacturer. Essentially, DNA was mixed with Fugene 6. After incubation at room temperature for 20 min, the mixture was directly added to the cells in a culture vessel. AAV-GFP was used as a control. Alternatively, electroporation (as described later) was used to introduce recombinant AAV DNA into ARPE-19 cells.

Human RPE cell line hTERT-RPE1 was purchased from Clontech. At 50% confluency, retrovirus MSCV-neuroD (5 ml) or MSCV-GFP as a control was added to the culture (75-cm^2^ flask). Polybrene (Sigma, St. Louis, MO) was added to a final concentration of 8 μg/ml to increase the efficiency of retroviral infection of the cell. After 4 h, the culture medium was replaced with 10 ml of D-MEM–F12+10% fetal calf serum (FCS; Life Technologies, Grand Island, New York, NY). Selection was performed using G418 (500 μg/ml, added to the culture medium) from day 3 through day 10. On day 10, the cells were reseeded into a 24-well plate and cultured for 2 days before fixation for analysis. Alternatively, the cells were electroporated (as described in the next paragraph) with recombinant MSCV DNA.

For electroporation, cells in an 80% confluent culture were harvested by trypsinization and centrifugation at 600 × *g* for 3 min at room temperature. Cells from a 25-cm^2^ flask were resuspended into 0.5 ml of PBS (137 mM NacCl, 2.7 mM KCl, 10 mM Na_2_HPO_4_, 2.0 mM KH_2_HPO_4_, pH 7.4). Recombinant DNA (5–10 µg in 20 µl of TE) was added to a 0.4-ml cell suspension, and the mixture was placed on ice for 10 min. Electorporation was performed in a cuvette with a 2-mm gap at 450 v for 75 µs, repeating twice with a 100-ms interval, using a BTX 830 Electro Square Porator (Harvard Apparatus, Boston, MA). After mixing by pipetting twice, the electroporation was repeated once. Culture medium (D-MEM–F12+10% FCS) was added, and the cells were seeded into four wells of a 12-well plate. Cells were cultured with D-MEM–F12+10% FCS for 10–14 days with the medium changed every other day.

### Primary RPE cell culture

The care and use of animals adhered to the procedures and policies published by the US Public Health Service (Public Health Service Police on Humane Care and Use of Animals) and set by the Institutional Animal Use and Care Committee at the University of Alabama at Birmingham. To establish a primary cell culture of mouse RPE, eyes were enucleated at postnatal day 5 (P5) after euthanasia by cervical dislocation followed by decapitation. Eyes were placed into a 60-mm dish with ice-cold L-15 medium supplemented with 10% FCS. After removing extraocular muscle and fat tissues with a pair of forceps, the eyeballs were placed into another 60-mm dish with ice-cold L-15+10% FCS. A circumferential incision was made along the ora serrata, and the cornea, the iris, the lens, and the vitreous were removed. The neural retina was then gently and completely removed. The remaining eyecup, sclera+choroid+RPE, was transferred into a 35-mm dish with ice-cold L-15+10% FCS and inspected for being free of residual retinal tissue. The RPE was then gently peeled off and placed into a 35-mm dish with ice-cold L-15+10% FCS. The isolated RPE tissue was transferred into a 15-ml tube, rinsed twice with calcium-magnesium-free medium, and treated with typsin/EDTA at 37 °C for 10 min. After centrifugation at 900 × *g* for 5 min at room temperature, the cells were resuspended in 1 ml of L-15+10% FCS and seeded into a 24-well plate (0.25 ml cells/well containing 0.5 ml of L-15+10% FCS). On the second day, medium was replaced with 0.5 ml of knockout D-MEM (Life Technologies) supplemented with 20% serum replacement (KO/SR). When the culture reached ~50% confluency, Lvx-ngn1-IRES-ZsGreen1 in the packaging cell culture medium without further concentration was added; the culture was then maintained for another 10–14 days before fixation for analysis.

Porcine eyes were obtained from animals after a cardiology study. Briefly, animals (3–6 months) were anesthetized by intramuscular injection of Telazol (4.4 mg/kg), xylazine (2.2 mg/kg), and atropine (0.04 mg/kg). Anesthesia was maintained with isoflurane in 100% oxygen by inhalation. Core body temperature, arterial blood pressure, arterial blood gases, and serum electrolytes were monitored and maintained within normal ranges throughout the electrophysiological study. ECG lead II was continuously displayed. Once data collection was completed, the animal was euthanized by an intravenous injection of potassium chloride while still at a surgical plane of anesthesia. Eyes were enucleated immediately after euthanasia. RPE was isolated from freshly enucleated eyes following the procedure of Grisanti and Guidry [19]. Briefly, RPE was dissected out of enucleated porcine eyes, and the isolated RPE tissue was treated with trypsin/EDTA. The dissociated cells were subjected to density gradient centrifugation through a cushion of 40% Percol to remove other (contaminating) cells [19]. Cells from two eyes were seeded into a six-well plate in knockout D-MEM supplemented with 10% FCS for 5–7 days to reach confluency. At that point, cells in the culture were trypsinized, reseeded at a 1:2 ratio into a 24-well plate, and cultured with KO/SR. When the culture was about 50% confluent, Lvx-ngn3-IRES-ZsGreen1 in the packaging cell culture medium (without concentration) was added; the culture was then maintained for 7–14 days before fixation for analysis.

### Immunocytochemistry

Cultured cells were fixed with ice-cold 4% paraformaldehyde in phosphate buffer for 30-60 min, followed by washing with PBS and incubating with primary antibody and then secondary antibody. Primary antibodies included monoclonal antibodies against Map2 (Sigma) and against rhodopsin (Millipore, Billerica, MA); polyclonal antibodies against arrestin (ABR Affinity BioReagents, Golden, CO), against transducin β-subunit (Affinity Bioreagents), against recoverin (Chemicon), and against red opsin (Millipore).

## Results

### Reprogramming cultured human cells

**Figure 1:**
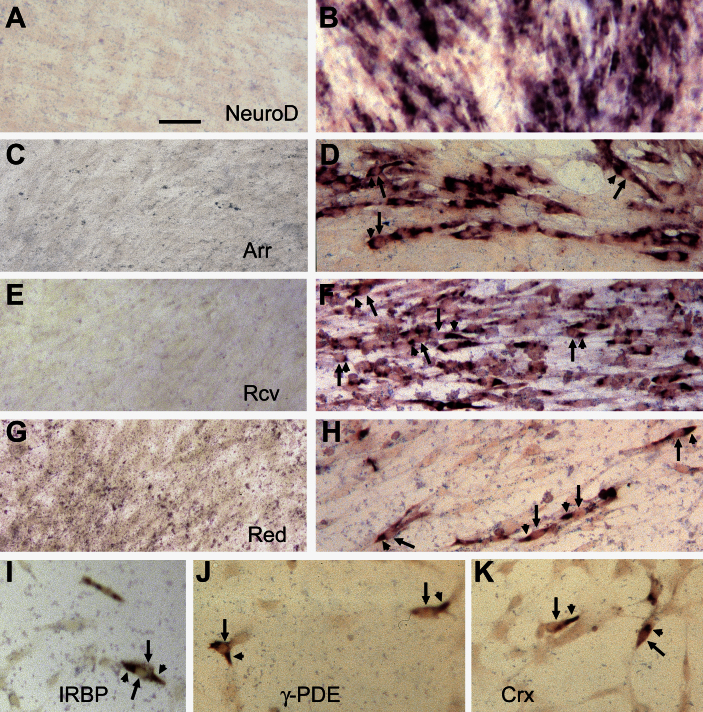
Detection photoreceptor gene expression in cultures of hTERT-RPE1 cells infected with retroviral MSCV expressing control GFP or experimental gene *neuroD*. Shown are in situ mRNA hybridization for *neuroD* mRNA (**A**, **B**), retinal cone arrestin 3 mRNA (*Arr*; **C**, **D**), recoverin mRNA (*Rcv*, **E**, **F**), and red opsin mRNA (*Red*; **G**, **H**), in the two sets of cultures with **A**, **C**, **E**, **G** representing the control. I-K are in situ hybridization detection for mRNA of interphotoreceptor retinoid binding protein (*IRBP*; **I**), the γ-subunit of phosphodiesterase (*γ-PDE*; **J**), and cone-rod homeobox protein (*Crx*; **K**) of hTERT-RPE1 cell culture infected with MSCV retrovirus expressing *neuroD*. Scale bar (20 µm) applies to all panels.

In the first set of experiments, we used hTERT-RPE1 cells and retrovirus MSCV expressing *neuroD*, the first gene identified with the chick system to be able to elicit photoreceptor differentiation in primary cell cultures of embryonic chicken RPE [20]. Transduction of hTERT-RPE1 cells by MSCV-neuroD was examined with in situ hybridization detection of *neuroD* mRNA. While the control culture infected with MSCV-GFP lacked cells positive for *neuroD* mRNA (Figure 1A), such positive cells were present in the experimental culture (Figure 1B). Transduction efficiency varied among different experiments, likely due to variations in the stage of culture (hence the stage of cell growth) at the time of viral administration. In the best case, cells positive for *neuroD* mRNA accounted for ~50% of the cells present. Both control and experimental cultures were then examined for the expression of photoreceptor genes encoding retinal cone arrestin-3 (Arr), recoverin (Rcv), red opsin (Red), interphotoreceptor retinoid binding protein (IRBP), the γ-subunit of phosphodiesterase (γ-PDE), and cone-rod homeobox protein (Crx). While expression of these genes was absent in the control (Figure 1C,E,G), cells expressing these genes were detected in the experimental culture (Figure 1D,F,H-K) at estimated frequencies of ~30% for Arr and Rcv, ~10% for IRBP and γ-PDE, and 5% for Crx and Red in cultures with ~50% transduction efficiency. Some of the positive cells displayed morphology reminiscent of young photoreceptor cells, with an elongated cell body (arrow) and an apex of cytoplasm decorated by in situ hybridization signals (arrowhead).

**Figure 2:**
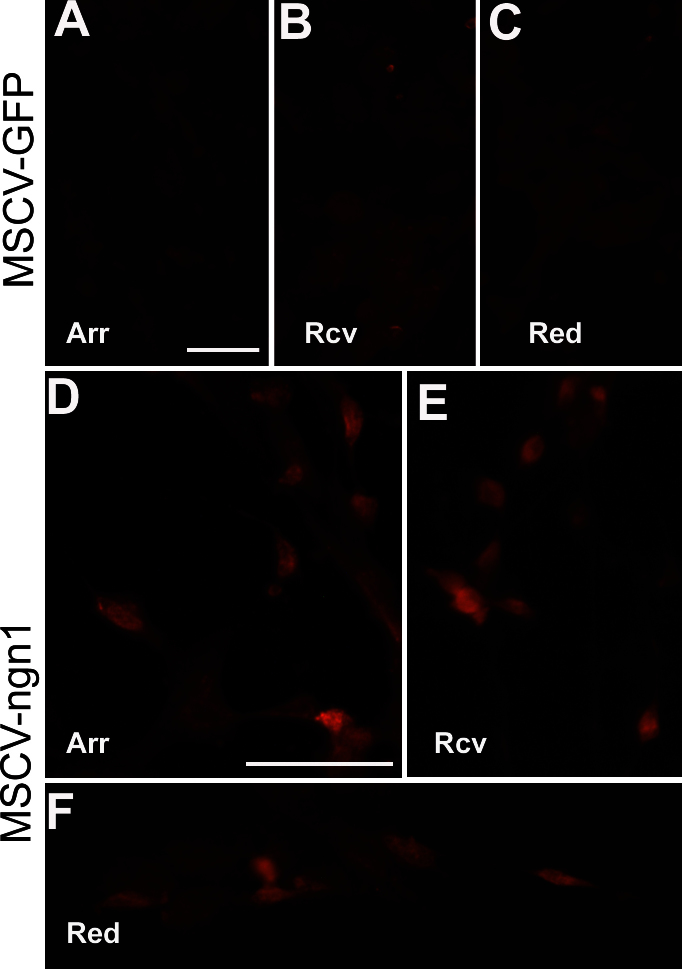
Expression of photoreceptor proteins in hTERT-RPE1 cultures transfected (by electroporation) with MSCV retroviral DNA expressing control GFP or experimental gene human *neurogenin1*. Shown are immunofluorescent detection in the control cultured transfected with MSCV DNA expressing control GFP (MSCV-GFP) for retinal cone arrestin 3 (Arr; **A**), recoverin (Rcv; **B**), and red opsin (Red; **C**) and in the experimental culture transfected with MSCV DNA expressing human *neurogenin1* (MSCV-ngn1) of each protein (**D**-**F**). Scale bars are 50 μm.

In subsequent experiments, hTERT-RPE1 cells were transfected with MSCV-ngn1 DNA through electroporation. Immunostaining showed the presence of cells positive for retinal cone arrestin 3 (Figure 2D, ~50% in a highly transduced area), recoverin (Figure 2E, ~50%), and red opsin (Figure 2F, ~15%), while the control (electroporated with MSCV-GFP) lacked such positive cells (Figure 2A-C).

**Figure 3:**
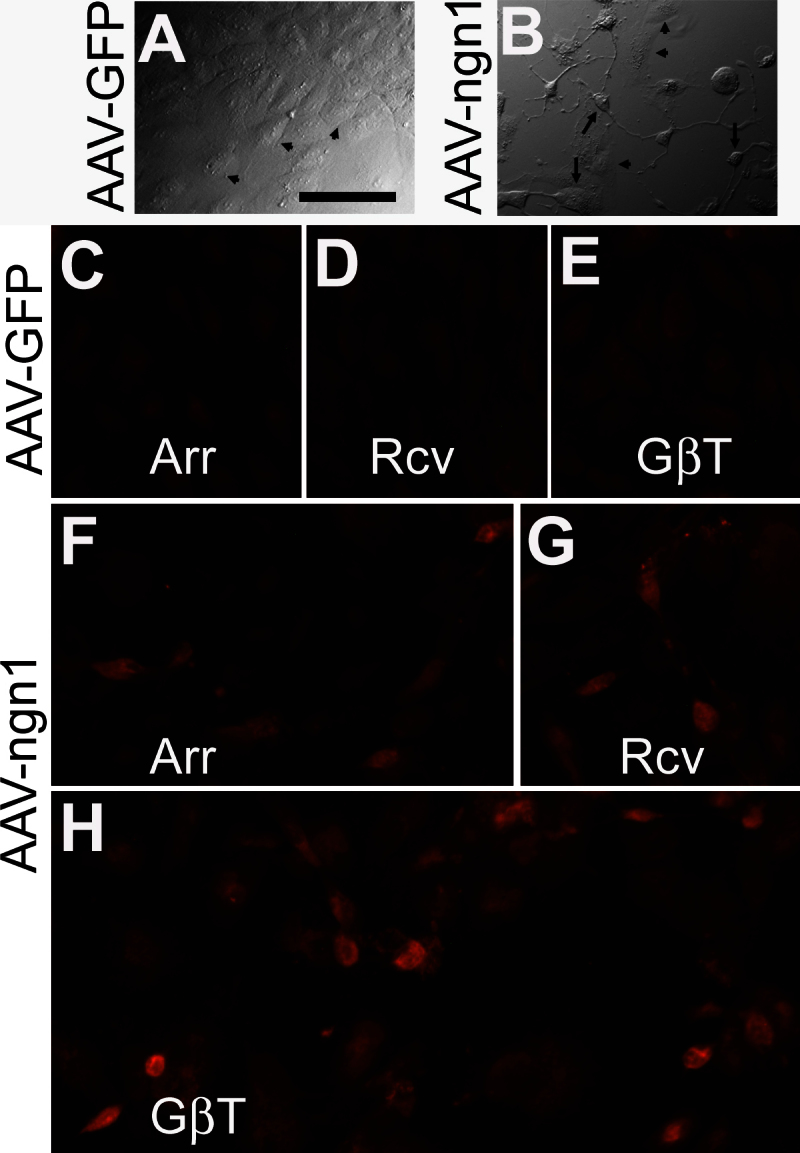
Photoreceptor-like cells in ARPE-19 cultures transfected with adeo-associated viral DNA expressing human *neurogenin1*. Shown are morphologies of cells in a control culture transfected with adeo-associated viral (AAV) DNA expressing GFP (AAV-GFP; **A**) and in an experimental culture transfected AAV DNA expressing human *neurogenin1* (AAV-ngn1: **B**). Arrows point to cells with neuron-like morphologies, and arrowheads point to cells maintaining the morphologies of ARPE-19 cells. **C**-**H** are immunofluorescent detection of photoreceptor proteins arrestin (Arr), recoverin (Rcv) and transducin β-subunit (GβT) in ARPE-19 cell cultures transfected with AAV-GFP (A-C) or AAV-ngn1 (**D**-**F**). Scale bar (50 μm) applies to all panels.

Because the retroviral vector is considered less compatible than the AAV vector for possible clinical application and hTERT-RPE1 is less RPE-like than ARPE-19, we then tested whether AAV-ngn1 would guide ARPE-19 cells to the path of differentiation toward photoreceptor cells. ARPE-19 cells were transfected using Fugen6 with recombinant AAV-ngn1 DNA. Three days after the introduction of AAV-ngn1 DNA, some cells began to exhibit neuronal morphologies (Figure 3B), while cells in the AAV-GFP DNA control maintained their typical flat appearance (Figure 3A). Further examination with immunostaining detected cells positive for arrestin (~10% in the best case), recoverin (~10%), and transducin β-subunit (~10%) in cultures treated with AAV-ngn1 (Figure 3F-H) but not with AAV-GFP (Figure 3C-E).

### Reprogramming mouse and porcine RPE cells

**Figure 4:**
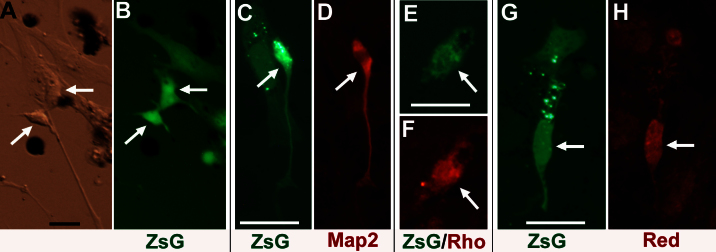
Photoreceptor-like cells in a postnatal day 5 mouse RPE cell culture infected with lenti virus Lvx-ngn1-IRES-ZsGreen1. Shown are bright field (**A**), epi-fluorescence for ZsGreen1 (**B**, **C**, **E**, **G**), and immunodetection for microtubule-associate protein 2 (Map2; **D**), rhodopsin (Rho; **F**), and red opsin (Red; **H**). Photoreceptor-like cells are indicated by arrows. Scale bars are 25 μm.

As an initial test of the possibility that the RPE in the mammalian eye can be deployed to regenerate photoreceptor cells to repopulate the retina without cell transplantation, we examined whether cells in a primary RPE cell culture of mouse and pig were responsive to the reprogramming scheme. Lvx-ngn1-IRES-ZsGreen1 was added to the primary cell culture of RPE isolated from P5 mice. Transduction by the lenti virus was monitored by viewing ZsGreen1 with epi-fluorescent microscopy. Three days after viral administration, some ZsGreen1^+^ cells (~50%), particularly those with brighter fluorescence (likely to have higher levels of transgene expression), began to exhibit morphologies resembling differentiating photoreceptor cells (Figure 4A,B, arrows). As the culture aged, the ratio of ZsGreen1^+^ cells with neural morphologies to ZsGreen1^+^ cells maintaining RPE morphologies decreased, likely due to the latter out-proliferating the former. Immunochemistry showed that some ZsGreen1^+^ cells were positive for neural maker Map2 (Figure 4C,D), rhodopsin (Figure 4E,F, ~20% of ZsGreen1^+^ cells in the four best viewing areas), or red opsin (Figure 4G,H, 20% of ZsGreen1^+^ cells in the six best viewing areas). Morphologically, ~30% of the reprogrammed cells appeared elongated and polarized, with a short apex and a thin long process on the basal side, morphology reminiscent of differentiating photoreceptor cells; 0% of the cells in the nonprimary human RPE cell cultures had this morphology (Figure 1, Figure 2, and Figure 3).

**Figure 5:**
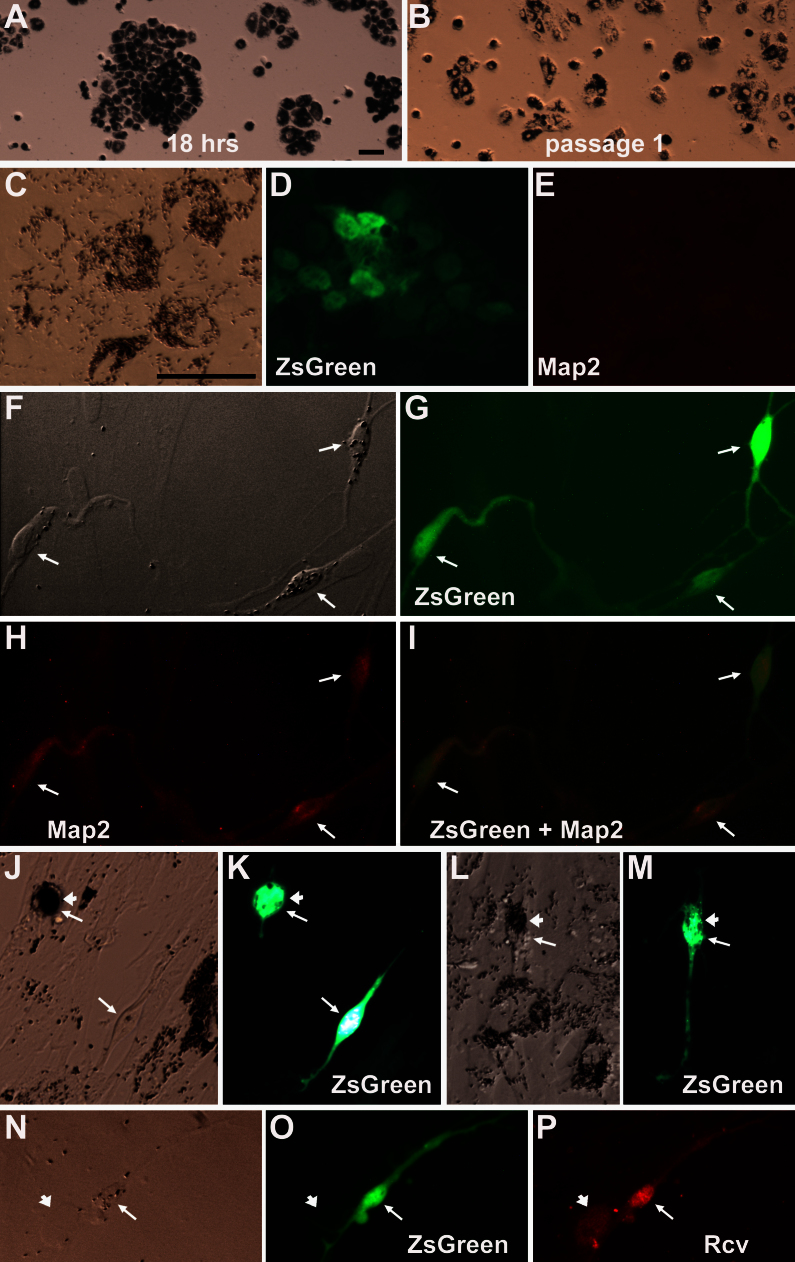
Photoreceptor-like cells in porcine RPE cell culture. **A**, **B** are bright field views of porcine RPE cells after 18 h in culture (**A**) and in a passage 1 culture (**B**). **C**-**E** are a control culture infected with lenti virus control, Lvx-IRES-ZsGreen1, under with bright-field (**C**), epi-fluorescence for ZsGreen1 (**D**), or immunostaining for microtubule-associate protein 2 (**E**). **F**-**I** are porcine RPE culture infected with lenti virus Lvx-ngn3-IRES-ZsGreen1 (expressing neurogenin3) under with bright-field (**F**), epi-fluorescence for ZsGreen1 (**G**), immunostaining for Map2 (**H**), or a merged view (**I**). Arrows point to ZsGreen1^+^ cells with a neural morphology. **J**-**M** show neuron-like ZsGreen1^+^ cells (arrow) with aggregate of dark pigment granules (arrowhead) in cultures infected with Lvx-ngn3-IRES-ZsGreen1. **N**-**P** are porcine RPE culture infected with Lvx-ngn1-IRES-ZsGreen1 under with bright-field (N), epi-fluorescence for ZsGreen1 (**O**), immunostaining for recoverin (**P**). Arrow points to a ZsGreen1^+^/Recoverin^+^ cell; arrowhead points to a ZsGreen1¯/Recoverin¯ cell. The scale bars are 25 µm and the one in **C** applies to all except **A**, **B**.

We then tested whether RPE cells from a large mammal (3–6-month-old pig) were responsive to the reprogramming scheme. To obtain a sufficient amount of high quality RPE cells with a limited number of eyes, the primary cell culture of porcine RPE (Figure 5A) was amplified once to produce a passage 1 culture (Figure 5B). The passage 1 culture was then subjected to reprogramming by adding Lvx-ngn3-IRES-ZsGreen1. While maintaining typical morphologies in the control (Figure 5C-E), ZsGreen1^+^ cells in the experimental culture exhibited morphology suggestive of differentiating photoreceptor cells (Figure 5G,K,M,O, arrow) and expressed Map2 (Figure 5H) and recoverin (Figure 5P). Notably, some ZsGreen1^+^ cells with neural morphology retained the dark pigment granules (Figure 5K,M, arrowhead) of the RPE.

## Discussion

Studies with the chick system raised the possibility of reprogramming RPE cells with a pro-photoreceptor gene for the generation of cells bearing photoreceptor traits. Nonetheless, such studies often lack high clinical relevance because the chick, a nonmammalian model, is evolutionally more ancient and may manifest phenotypical changes that are lacking in a mammal after comparable experimental manipulations. To move forward toward the ultimate therapeutic goal of inducing in situ photoreceptor regeneration from ocular tissue, specifically the RPE, we studied mammalian cells, including human cells. Obviously, primary human RPE cells derived from donated eyes would be ideal for testing biologic feasibility. However, because of limited availability of donated eyes and because of ample availability of alternative cell sources, we included two human RPE cell lines, hTERT-RPE1 and ARPE-19. hTERT-RPE1 was used for its RPE origin and its potential as an unlimited cell source. hTERT-RPE1 is a telomerase-immortalized RPE cell line derived from a 1-year-old child by Geron Corporation (Menlo Park, CA). hTERT-RPE1 cells can divide indefinitely, retain some RPE phenotypes, and are not tumorigenic [26]. ARPE-19 is a spontaneously arising RPE cell line derived by Amy Aotaki-Keen (University of California, Davis, CA) from the normal eyes of a 19-year-old individual. Unlike hTERT-RPE1 cells, which can divide indefinitely, ARPE-19 cells can be subcultured to a limited number of passages. Cells in ARPE-19 maintain some RPE properties, such as expression of RPE-specific markers CRALBP and RPE-65, formation of stable monolayers with morphological and functional polarity, and phagocytosis of shed photoreceptor rod outer segments [27,28].

The generation of cells reminiscent of young photoreceptor from hTERT-RPE1 and ARPE-19 cultures ectopically expressing *neuroD* or *ngn1* suggests that human cells can respond to the reprogramming modality by taking on the path of differentiating toward photoreceptor cells. The morphological resemblance of the new cells in an hTERT-RPE1 culture to that of differentiating photoreceptor cells seems rather remarkable considering that the immortalized cells may have substantially deviated from the original RPE. Despite the positive outcome, the two human cell lines may not be ideal sources of new photoreceptor cells. First, the high proliferative capability of the cell line cells may impede the progress of photoreceptor differentiation in those reprogrammed cells. Second, conflict between cell proliferation and photoreceptor differentiation may induce or promote the death program in reprogrammed cells.

In lieu of human tissue, mouse and porcine RPE were isolated and used to establish primary cell cultures in order to supplement experiments with hTERT-RPE1 and ARPE-19 cell lines. Introduction of *ngn1* or *ngn3* resulted in the primary RPE cells changing gene expression and cellular morphologies, suggesting that they are responsive to the reprogramming. The presence of pigment granules in cells with photoreceptor-like morphology implies a direct “transdifferentiation” from RPE to photoreceptor-like cells. The reprogrammed cells’ polarized cell body, implicative of differentiating photoreceptors, might reflect an advantage of primary RPE over cell line cells with respect to allowing the advancement of photoreceptor differentiation. Alternatively, it may reflect an advantage of primary RPE over cell line cells with respect to providing a supportive environment for advanced photoreceptor differentiation of other cells, or even itself. Regardless, this arguably favors the prospect of producing mature photoreceptor cells from reprogramming mammalian RPE in situ in an adult eye.
